# Supplementary material for: Quality of life after resection of a meningioma—A cross-cultural comparison of Indian and Australian patients
Source: PLoS One. 2022 Sep 26;17(9):e0275184. doi: 10.1371/journal.pone.0275184 (PMC9512203; doi:10.1371/journal.pone.0275184)
Supplement: S1 Table — (DOCX) [file pone.0275184.s002.docx]

## Appendix 1

**QLQ-C30 General health status and functional scales**

| Time | Australia^  (mean) | India^  (mean) | Mean diff.^^ | Lower 95% CI of mean diff. | Upper 95% CI of mean diff. | Mixed model analysis with interaction effect (p-values)^^^ | | |
| --- | --- | --- | --- | --- | --- | --- | --- | --- |
|  |  |  |  |  |  | Country x time | time | country |
| Global QoL | | | | | | | | |
| T1 | 66.6 | 64.6 | 2.0 | -5.5 | 10.0 | <.001 | <.001 | 0.022 |
| T2 | 70.0 | 70.8 | -0.7 | -8.8 | 7.4 |  |  |  |
| T3 | 69.1 | 77.3 | -8.1 | -18.7 | 2.5 |  |  |  |
| T4 | 68.0 | 88.2 | -20.3* | -29.7 | -10.8 |  |  |  |
| Physical functioning | | | | | | | | |
| T1 | 82.9 | 63.1 | 19.8* | 10.4 | 29.2 | 0.127 | 0.005 | 0.006 |
| T2 | 85.2 | 72.7 | 12.5.* | 2.4 | 22.6 |  |  |  |
| T3 | 87.3 | 78.3 | 8.9 | -4.9 | 22.7 |  |  |  |
| T4 | 86.0 | 81.2 | 4.8 | -7.6 | 17.2 |  |  |  |
| Role functioning | | | | | | | | |
| T1 | 59.0 | 68.7 | -9.8* | -19.3 | -0.3 | 0.098 | <.001 | 0.836 |
| T2 | 81.7 | 75.0 | 6.6 | -3.9 | 17.1 |  |  |  |
| T3 | 78.5 | 81.5 | -3.0 | -17.5 | 11.5 |  |  |  |
| T4 | 84.9 | 81.6 | 3.3 | -10.3 | 16.8 |  |  |  |
| Emotional functioning | | | | | | | | |
| T1 | 71.6 | 64.8 | 6.8 | -2.8 | 16.3 | 0.012 | 0.233 | 0.987 |
| T2 | 76.7 | 68.7 | 8.0 | -2.5 | 18.4 |  |  |  |
| T3 | 76.0 | 75.0 | 1.0 | -13.0 | 15.1 |  |  |  |
| T4 | 66.0 | 81.6 | -15.6* | -28.7 | -2.5 |  |  |  |
| Cognitive functioning | | | | | | | | |
| T1 | 72.3 | 73.5 | -1.2 | -9.8 | 7.5 | 0.319 | 0.729 | 0.065 |
| T2 | 74.6 | 75.9 | -1.3 | -10.7 | 8.1 |  |  |  |
| T3 | 71.3 | 82.1 | -10.8 | -23.5 | 1.9 |  |  |  |
| T4 | 70.0 | 80.7 | -10.6 | -22.4 | 1.1 |  |  |  |
| Social functioning | | | | | | | | |
| T1 | 68.2 | 76.3 | -8.2 | -18.1 | 1.7 | 0.942 | 0.039 | 0.021 |
| T2 | 74.2 | 82.0 | -7.8 | -18.6 | 3.1 |  |  |  |
| T3 | 76.7 | 88.6 | -11.9 | -26.9 | 3.1 |  |  |  |
| T4 | 79.1 | 84.8 | -5.8 | -19.8 | 8.3 |  |  |  |
| * indicates a statistically significant difference at p ≤ 0.05  ^ N for Australia at T1 = 49, T2 = 38, T3 = 29, T4 = 68,  N for India at T1 = 57, T2 = 50, T3 = 17, T4 = 14  ^^ numbers may not add up due to rounding  ^^^ p-values from type III tests of fixed effects | | | | | | | | |
