# Supplementary material for: Quality of life after resection of a meningioma—A cross-cultural comparison of Indian and Australian patients
Source: PLoS One. 2022 Sep 26;17(9):e0275184. doi: 10.1371/journal.pone.0275184 (PMC9512203; doi:10.1371/journal.pone.0275184)
Supplement: S2 Table — (DOCX) [file pone.0275184.s003.docx]

## Appendix 2

**QLQ-C30 symptom scales**

| Time | Australia^  (mean) | India^  (mean) | Mean diff.^^ | Lower 95% CI of mean diff. | Upper 95% CI of mean diff. | Mixed model analysis with interaction effect (p-values)^^^ | | |
| --- | --- | --- | --- | --- | --- | --- | --- | --- |
|  |  |  |  |  |  | Country x time | time | country |
| Fatigue | | | | | | | | |
| T1 | 40.3 | 29.7 | 10.6* | 1.9 | 19.2 | 0.224 | <.001 | 0.062 |
| T2 | 25.4 | 25.3 | 0.1 | -9.5 | 9.6 |  |  |  |
| T3 | 27.2 | 15.4 | 11.8 | -1.2 | -24.7 |  |  |  |
| T4 | 26.5 | 24.2 | 2.3 | -9.6 | -14.1 |  |  |  |
| Nausea and Vomiting | | | | | | | | |
| T1 | 9.3 | 15.7 | -6.4* | -11.4 | -0.3 | 0.178 | 0.011 | 0.031 |
| T2 | 4.1 | 14.5 | -10.4* | -17.1 | -3.6 |  |  |  |
| T3 | 3.3 | 7.9 | -4.6 | -13.8 | 4.6 |  |  |  |
| T4 | 5.9 | 5.5 | -0.4 | -8.0 | 8.8 |  |  |  |
| Pain | | | | | | | | |
| T1 | 24.4 | 29.2 | -4.8 | -14.6 | 4.9 | 0.543 | 0.261 | 0.981 |
| T2 | 23.1 | 26.0 | -2.9 | -13.6 | 7.9 |  |  |  |
| T3 | 25.7 | 21.5 | 4.2 | -10.2 | 18.6 |  |  |  |
| T4 | 21.3 | 17.5 | 3.9 | -9.3 | 17.0 |  |  |  |
| Dyspnoea | | | | | | | | |
| T1 | 13.0 | 21.1 | -8.1 | -17.0 | 0.7 | 0.110 | 0.303 | 0.389 |
| T2 | 12.9 | 19.2 | -6.4 | -16.3 | 3.5 |  |  |  |
| T3 | 16.1 | 21.5 | -5.4 | -18.6 | 7.8 |  |  |  |
| T4 | 15.6 | 7.7 | 8.0 | -4.1 | 19.9 |  |  |  |
| Insomnia | | | | | | | | |
| T1 | 44.3 | 38.7 | 5.6 | -6.2 | 17.4 | 0.244 | 0.001 | 0.196 |
| T2 | 25.8 | 27.7 | 1.9 | -15.0 | 11.1 |  |  |  |
| T3 | 34.8 | 15.8 | 19.0* | 1.1 | 36.9 |  |  |  |
| T4 | 36.3 | 36.0 | 0.3 | -16.2 | 16.8 |  |  |  |
| Appetite loss | | | | | | | | |
| T1 | 18.6 | 33.0 | -14.4* | -23.3 | -5.5 | 0.313 | 0.001 | 0.025 |
| T2 | 12.2 | 21.4 | -9.2 | -19.0 | 0.6 |  |  |  |
| T3 | 17.2 | 21.7 | -4.5 | -18.0 | 9.0 |  |  |  |
| T4 | 10.2 | 11.8 | -1.6 | -14.1 | 14.1 |  |  |  |
| Constipation | | | | | | | | |
| T1 | 11.8 | 19.1 | -1.3 | -10.7 | 8.0 | 0.442 | 0.016 | 0.291 |
| T2 | 12.7 | 19.2 | -6.5 | -16.8 | 3.7 |  |  |  |
| T3 | 4.2 | 14.4 | -10.2 | -24.4 | 4.0 |  |  |  |
| T4 | 9.3 | 6.0 | 3.4 | -9.7 | 16.5 |  |  |  |
| Diarrhoea | | | | | | | | |
| T1 | 8.7 | 14.8 | -6.2 | -12.9 | 0.6 | 0.075 | 0.213 | 0.011 |
| T2 | 2.1 | 17.4 | -15.3* | -23.0 | -7.6 |  |  |  |
| T3 | 5.1 | 7.5 | -2.4 | -13.9 | 8.1 |  |  |  |
| T4 | 5.8 | 8.3 | -2.4 | -12.3 | 7.4 |  |  |  |
| Financial difficulties | | | | | | | | |
| T1 | 29.1 | 27.4 | -1.7 | -9.7 | 13.0 | 0.862 | 0.148 | 0.757 |
| T2 | 23.6 | 25.6 | -2.0 | -14.6 | 10.6 |  |  |  |
| T3 | 26.2 | 27.6 | -1.1 | -18.8 | 16.7 |  |  |  |
| T4 | 20.1 | 13.7 | 6.5 | -10.1 | 23.0 |  |  |  |
| * indicates a statistically significant difference at p ≤ 0.05  ^ N for Australia at T1 = 49, T2 = 38, T3 = 29, T4 = 68,  N for India at T1 = 57, T2 = 50, T3 = 17, T4 = 14  ^^ numbers may not add up due to rounding  ^^^ p-values from type III tests of fixed effects | | | | | | | | |
