# Supplementary material for: Quality of life after resection of a meningioma—A cross-cultural comparison of Indian and Australian patients
Source: PLoS One. 2022 Sep 26;17(9):e0275184. doi: 10.1371/journal.pone.0275184 (PMC9512203; doi:10.1371/journal.pone.0275184)
Supplement: S3 Table — (DOCX) [file pone.0275184.s004.docx]

## Appendix 3

**BN20 scales**

| Time | Australia^  (mean) | India^  (mean) | Mean diff.^^ | Lower 95% CI of mean diff. | Upper 95% CI of mean diff. | Mixed model analysis with interaction effect (p-values)^^^ | | |
| --- | --- | --- | --- | --- | --- | --- | --- | --- |
|  |  |  |  |  |  | Country x time | time | country |
| Future uncertainty | | | | | | | | |
| T1 | 23.6 | 22.9 | 0.7 | -7.9 | 9.3 | 0.348 | 0.335 | 0.094 |
| T2 | 26.3 | 21.1 | 5.2 | -4.0 | 14.3 |  |  |  |
| T3 | 29.0 | 17.2 | 11.7* | 0.2 | 23.2 |  |  |  |
| T4 | 22.3 | 16.0 | 6.4 | -3.8 | 16.6 |  |  |  |
| Visual disorder | | | | | | | | |
| T1 | 24.5 | 12.8 | 11.8* | 4.2 | 19.2 | 0.941 | 0.209 | <.001 |
| T2 | 20.9 | 9.8 | 11.1* | 2.8 | 19.4 |  |  |  |
| T3 | 22.5 | 9.8 | 12.7* | 2.1 | 23.2 |  |  |  |
| T4 | 17.9 | 8.8 | 9.1 | -0.4 | 18.6 |  |  |  |
| Motor dysfunction | | | | | | | | |
| T1 | 10.6 | 3.8 | 6.7* | 0.7 | 12.8 | 0.145 | 0.374 | 0.001 |
| T2 | 8.1 | 1.8 | 6.2 | -0.3 | 12.6 |  |  |  |
| T3 | 10.9 | 0 | 10.9* | 3.3 | 18.5 |  |  |  |
| T4 | 11.0 | -1.4 | 12.4* | 5.6 | 19.1 |  |  |  |
| Communication deficit | | | | | | | | |
| T1 | 17.7 | 3.6 | 14.1* | 7.4 | 20.8 | 0.129 | 0.543 | <.001 |
| T2 | 11.0 | 5.0 | 6.0 | -1.0 | 13.1 |  |  |  |
| T3 | 14.9 | 3.8 | 11.1* | 2.7 | 19.5 |  |  |  |
| T4 | 15.8 | 3.1 | 12.7* | 5.2 | 20.2 |  |  |  |
| Headaches | | | | | | | | |
| T1 | 37.4 | 12.3 | 25.1* | 6.1 | 44.0 | 0.067 | 0.146 | 0.002 |
| T2 | 19.7 | 13.9 | 5.8 | -14.8 | 26.5 |  |  |  |
| T3 | 43.0 | 4.7 | 38.3* | 14.4 | 62.2 |  |  |  |
| T4 | 30.4 | 3.9 | 26.5* | 3.5 | 49.5 |  |  |  |
| Seizures | | | | | | | | |
| T1 | 2.8 | 3.1 | -0.3 | -3.3 | 2.7 | 0.174 | 0.933 | 0.792 |
| T2 | 1.4 | 4.7 | -3.3 | -6.7 | 0.1 |  |  |  |
| T3 | 4.5 | 2.5 | 2.1 | -2.5 | 6.6 |  |  |  |
| T4 | 3.8 | 3.5 | 0.3 | -3.8 | 4.5 |  |  |  |
| Drowsiness | | | | | | | | |
| T1 | 27.8 | 8.9 | 18.9* | 9.7 | 28.1 | 0.233 | 0.073 | <.001 |
| T2 | 18.1 | 10.4 | 7.7 | -2.2 | 17.6 |  |  |  |
| T3 | 16.6 | 6.9 | 9.8 | -3.0 | 22.5 |  |  |  |
| T4 | 18.6 | 3.1 | 15.6* | 4.2 | 27.0 |  |  |  |
| Itchy skin | | | | | | | | |
| T1 | 16.8 | -0.1 | 17.6* | 8.2 | 27.1 | 0.897 | 0.861 | <.001 |
| T2 | 21.6 | -0.1 | 22.3* | 11.6 | 33.1 |  |  |  |
| T3 | 17.1 | -1.3 | 18.5* | 4.0 | 32.9 |  |  |  |
| T4 | 19.5 | -0.1 | 20.2* | 7.4 | 33.1 |  |  |  |
| Hair loss | | | | | | | | |
| T1 | 24.2 | 5.5 | 18.7* | 11.1 | 32.6 | 0.018 | 0.017 | 0.001 |
| T2 | 16.1 | 5.6 | 10.5* | 2.6 | 25.0 |  |  |  |
| T3 | 13.8 | 5.7 | 8.1 | -1.2 | 22.9 |  |  |  |
| T4 | 12.8 | 5.3 | 7.5 | -0.9 | 20.8 |  |  |  |
| Weakness of legs | | | | | | | | |
| T1 | 11.0 | 5.6 | 5.3 | -1.1 | 11.8 | 0.919 | 0.217 | 0.179 |
| T2 | 8.0 | 3.9 | 4.1 | -3.1 | 11.3 |  |  |  |
| T3 | 4.8 | 2.3 | 2.5 | -7.1 | 12.0 |  |  |  |
| T4 | 5.7 | 3.2 | 2.5 | -6.0 | 11.0 |  |  |  |
| Bladder control | | | | | | | | |
| T1 | 12.3 | 5.9 | 6.5* | 0.2 | 12.7 | 0.434 | 0.133 | 0.007 |
| T2 | 15.4 | 7.4 | 8.0* | 1.5 | 14.4 |  |  |  |
| T3 | 14.8 | 6.8 | 8.0* | 0.9 | 15.0 |  |  |  |
| T4 | 16.5 | 6.4 | 10.1* | 3.6 | 16.6 |  |  |  |
| * indicates a statistically significant difference at p ≤ 0.05  ^ N for Australia at T1 = 49, T2 = 38, T3 = 29, T4 = 68, N for India at T1 = 57, T2 = 50, T3 = 17, T4 = 14  ^^ numbers may not add up due to rounding  ^^^ p-values from type III tests of fixed effects | | | | | | | | |
