# Supplementary material for: Quality of life after resection of a meningioma—A cross-cultural comparison of Indian and Australian patients
Source: PLoS One. 2022 Sep 26;17(9):e0275184. doi: 10.1371/journal.pone.0275184 (PMC9512203; doi:10.1371/journal.pone.0275184)
Supplement: S4 Table — (DOCX) [file pone.0275184.s005.docx]

**Appendix 5**

**Percentages of patients with anxiety and depression (assessed with HADS)**

Scoring:

Normal: 0-7

Borderline: 8-10

Abnormal: 11-21

Anxiety

|  |  | T1 | T2 | T3 | T4 |
| --- | --- | --- | --- | --- | --- |
|  |  | % | % | % | % |
| Australia | normal | 75 | 73.3 | 81.8 | 72.4 |
| India | normal | 81.1 | 74.4 | 75 | 84.6 |
| Australia | borderline | 10 | 13.3 | 18.2 | 20.7 |
| India | borderline | 9.4 | 16.3 | 6.3 | 15.4 |
| Australia | abnormal | 15 | 13.3 | 0 | 6.9 |
| India | abnormal | 9.4 | 9.3 | 18.8 | 0 |

Depression

|  |  | T1 | T2 | T3 | T4 |
| --- | --- | --- | --- | --- | --- |
|  |  | % | % | % | % |
| Australia | normal | 75 | 86.7 | 100 | 79.3 |
| India | normal | 77.4 | 83.7 | 94.1 | 84.6 |
| Australia | borderline | 10 | 0 | 0 | 17.2 |
| India | borderline | 3.8 | 7 | 5.9 | 15.4 |
| Australia | abnormal | 15 | 13.3 | 0 | 3.4 |
| India | abnormal | 18.9 | 9.3 | 0 | 0 |
