# Supplementary material for: Quality of life after resection of a meningioma—A cross-cultural comparison of Indian and Australian patients
Source: PLoS One. 2022 Sep 26;17(9):e0275184. doi: 10.1371/journal.pone.0275184 (PMC9512203; doi:10.1371/journal.pone.0275184)
Supplement: S5 Table — (DOCX) [file pone.0275184.s006.docx]

## Appendix 4

**Anxiety and depression scores**

|  | Australia^  (mean) | India^  (mean) | Mean diff.^^ | Lower 95% CI of mean diff. | Upper 95% CI of mean diff. | Mixed model analysis with interaction effect (p-values)^^^ | | |
| --- | --- | --- | --- | --- | --- | --- | --- | --- |
|  |  |  |  |  |  | Country x time | time | country |
| Anxiety | | | | | | | | |
| T1 | 6.9 | 7.3 | -0.3 | -2.5 | 1.8 | 0.984 | 0.845 | 0.653 |
| T2 | 7.2 | 7.3 | -0.2 | -2.5 | 2.2 |  |  |  |
| T3 | 6.7 | 7.4 | -0.8 | -3.7 | 2.1 |  |  |  |
| T4 | 7.6 | 7.8 | -0.2 | -2.5 | 2.2 |  |  |  |
| Depression | | | | | | | | |
| T1 | 4.5 | 4.8 | -0.2 | -2.1 | 1.7 | 0.513 | 0.212 | 0.795 |
| T2 | 3.7 | 4.0 | -0.3 | -2.4 | 1.8 |  |  |  |
| T3 | 3.0 | 3.1 | -0.2 | -2.8 | 2.4 |  |  |  |
| T4 | 4.6 | 3.1 | 1.5 | -0.7 | 3.6 |  |  |  |
| * indicates a statistically significant difference at p ≤ 0.05  ^ N for Australia at T1 = 49, T2 = 38, T3 = 29, T4 = 68,  N for India at T1 = 57, T2 = 50, T3 = 17, T4 = 14  ^^ numbers may not add up due to rounding  ^^^ p-values from type III tests of fixed effects | | | | | | | | |
