# Supplementary material for: Quality of life after resection of a meningioma—A cross-cultural comparison of Indian and Australian patients
Source: PLoS One. 2022 Sep 26;17(9):e0275184. doi: 10.1371/journal.pone.0275184 (PMC9512203; doi:10.1371/journal.pone.0275184)
Supplement: Attachment — Submitted filename: Responses to reviewers.docx [file pone.0275184.s007.docx]

Dear Reviewers and Editors,

We appreciate the reviewer feedback and understand the value of the time they took to review our manuscript. Their comments and suggestions have allowed us to improve our work. Please find below our responses to the review comments.

We have prepared our article according to journal guidelines and updated file names and the reference list. We completed the inclusivity in global research questionnaire, which can be accessed as a supplementary file. Figure files will be provided in the requested format if the manuscript is accepted for publication.

Thank you for considering this manuscript for publication.

Kind regards,

Verena Schadewaldt

| Reviewer 1 |  |
| --- | --- |
| 1: The limitation of the study should be written shorter. | We have shortened the limitations section. Please see track changes in the manuscript. |
| 2: The study Conclusion should be written shorter. 3: The conclusion of the study should be written shorter | We have shortened the conclusion section. Please see track changes in the manuscript. |
| 3: It is suggested that the results shown in the figure are given in the tables. | The results shown in the figures 1-3 are shown in the Tables 1-3. The means for each time point per group are presented in the figures, directly derived from the means reported in the Tables 1-3.  We added a Table presenting the percentages from Figure 4 as a supplemental file S4_Table |
|  |  |
| Reviewer 2 |  |
| The introduction is discussion too long. Please summarize this part while maintain the focus on the research question. | We have shortened the introduction section. Please see track changes in the manuscript. |
| The sentence" Additionally, accounting for international differences in health care systems, economic status and individual lifestyles that may affect HRQoL is generally not possible in systematic reviews" needs citation. | We deleted this sentence as part of the shortening process of the introduction section. |
| Method: Clearly define all outcomes, predictors, potential confounders, and effect modifiers (if any). Did the study have any missing data? How did you approach them? | We added some words to clarify these variables: “Independent factors (outcome variables) were HRQoL, symptom burden, anxiety and depression. Dependent factors (predictors) were time point and country.” P. 11  Confounding factors were already listed in the manuscript: “Potentially confounding factors such as age, gender, WHO grade, tumour location and lateralization, SES, employment status and relationship status were added to the model if they showed a significant association with the outcome variables.”p. 11  Missing data: We added a sentence in the data analysis section, explaining that there was more missing data for the Australia sample for the HADS:  “The HADS was introduced later to the Australian study, so fewer patients completed it, leading to some missing data.“ p. 9  In the data analysis section we added a sentence about missing data: “We had minimal missing data: Across all time points and survey items missing data were below 0.5% for the EORTC QLQ-C30 and BN20. Of those patients who completed EORTC surveys, there were 13 in the Indian sample that did not complete the HADS due fatigue or refusal to respond and 110 in the Australian sample who did not receive the HADS, leaving 138 (India) and 184 (Australia) surveys for analysis. There were no missing items on the available HADS surveys in both groups. LMM procedures prevent listwise deletion due to missing data, maximizing the use of available data.” p. 10 |
| Results  Add a table describing the mean and SD of all measures and related sub-scales according to time-point and groups of concern. | The means are presented for all measures and sub-scales by time point and group in the online supplementary files S1-S4. The SD is not presented as on advice of our statistician we opted to present the mean difference and confidence intervals. |
| The discussion section is too long. Please revise. | We have shortened the discussion section. Please see track changes in the manuscript. |
| Some references are too old, please replace with newer ones. The reference list should include references published over the last 5 years. | We attempted to include the most up to date literature. This includes literature that is more than 5 years old as some of the concepts, measures and the patient groups (meningioma patients) covered in this study are quite specific and therefore not much more recent literature has been identified. |
